# A systematic review and meta-analysis of postoperative intervention effects in elderly patients with gastrointestinal cancer based on the protection motivation theory

**DOI:** 10.3389/fonc.2025.1620186

**Published:** 2025-11-11

**Authors:** Yanhong Wei, Li Zhang, Zhenqi Wei, Li Zheng, Chen Chai, Ye Ding

**Affiliations:** 1 Department of Ophthalmology and Otorhinolaryngology, The People’s Hospital of Suzhou New District, Suzhou, Jiangsu, China; 2 Department of Nursing, The People’s Hospital of Suzhou New District, Suzhou, Jiangsu, China; 3 Department of District Joint surgery, The People’s Hospital of Suzhou New, Suzhou, Jiangsu, China; 4 Department of District General Surgery, The People’s Hospital of Suzhou New, Suzhou, Jiangsu, China

**Keywords:** protection motivation theory, postoperative intervention, elderly gastrointestinal cancer, quality of life, psychological stress

## Abstract

**Objective:**

This study aims to evaluate the effect of postoperative interventions based on the Protection Motivation Theory (PMT) on postoperative recovery in elderly gastrointestinal cancer patients, providing scientific evidence to support clinical practice and improving postoperative recovery quality and survival rates in elderly patients.

**Methods:**

A systematic review and meta-analysis were conducted, with relevant studies retrieved from multiple Chinese and English databases. The studies selected met the inclusion criteria, involving elderly gastrointestinal cancer patients aged 60 years or older, and interventions guided by PMT, including health education, behavior modification, or psychological interventions. The primary outcomes assessed were postoperative complication rates, psychological stress scores, and quality of life scores.

**Results:**

A total of 8 randomized controlled trials (RCTs) were included, with a total of 319 participants. Meta-analysis results indicated that the PMT intervention group had a significantly lower postoperative complication rate compared to the conventional health education group (MD = 1.81, 95% CI:1.30–2.53, *P* = 0.0005). The PMT intervention group also showed significantly lower postoperative psychological stress scores than the conventional group (MD = -15.64, 95% CI: -17.34 to -13.95, *P* < 0.00001). Moreover, the PMT intervention group exhibited significantly better postoperative quality of life scores compared to the conventional care group (MD=-8.99, 95% CI:-9.60 to-8.38, *P* < 0.00001).

**Conclusion:**

Postoperative interventions based on the Protection Motivation Theory can significantly improve postoperative recovery in elderly gastrointestinal cancer patients by enhancing recovery outcomes, reducing complication rates, improving psychological stress levels, and enhancing quality of life, demonstrating strong clinical application value.

## 1 Introduction

As the global population ages, the health challenges faced by the elderly are increasing, particularly in the field of cancer. Gastrointestinal tumors, as one of the most common malignant tumors, exhibit notably high incidence and mortality rates in the elderly population (1). According to global cancer statistics, the incidence of gastrointestinal tumors ranks among the highest across all age groups, and elderly patients face greater challenges in treatment and postoperative recovery due to factors such as declining physiological function and weakened immune systems (2). With the advancement of medical technology, surgery has become a common method for treating gastrointestinal tumors, but postoperative recovery remains a crucial factor affecting patients’ quality of life and survival (3). In recent years, as research has deepened, more studies have begun to focus on the impact of postoperative interventions on patients’ recovery. Increasing evidence suggests that postoperative interventions not only improve patients’ quality of life but also reduce the incidence of complications, thereby enhancing survival rates (4). However, although there has been some accumulation of research on postoperative interventions for elderly gastrointestinal cancer patients, systematic reviews and meta-analyses remain scarce. Therefore, this study aims to evaluate the effect of postoperative interventions based on Protection Motivation Theory (PMT) on the postoperative recovery of elderly gastrointestinal cancer patients, further providing scientific evidence for clinical practice.

Protection Motivation Theory (PMT), first proposed by Rogers in 1975, is designed to explain individuals’ behavioral responses when faced with health threats (5). According to this theory, when individuals face a health threat, they assess the severity of the threat, their susceptibility to it, and the effectiveness of available coping measures, which then influence their decision to engage in protective behaviors. The core concept of PMT is that an individual’s health behavior is driven by two factors: threat appraisal and coping appraisal. Threat appraisal involves the recognition of the severity and susceptibility of the health threat, while coping appraisal involves the evaluation of the effectiveness of the coping measures (6). Studies have shown that PMT provides a structured theoretical framework for designing behavioral interventions, not only for disease prevention but also broadly within disease treatment contexts. Its application is particularly relevant in postoperative rehabilitation, where improving patient adherence and facilitating health behavior change are crucial. Over the past few decades, PMT has been widely applied in various health behavior intervention fields, including smoking cessation, alcohol consumption, weight management, and exercise. Research has found that interventions based on PMT can significantly enhance individuals’ behavioral changes when facing health threats, such as increasing physical activity, improving dietary habits, and boosting vaccination rates (7). Particularly in the elderly population, the application of PMT has shown promising results, with many studies indicating that enhancing elderly patients’ cognitive awareness of postoperative recovery and self-efficacy can effectively improve their adherence to rehabilitation, thereby improving recovery outcomes. In the postoperative rehabilitation of elderly gastrointestinal cancer patients, factors such as psychological state, health cognition, and adherence play key roles in recovery. Postoperative patients often face multiple challenges, including physical recovery, dietary adjustments, and prevention of complications. In the early postoperative phase, patients may struggle to actively engage in rehabilitation activities due to pain, fatigue, and emotional distress (8). Therefore, this study will use systematic review and meta-analysis methods to integrate existing research findings and evaluate the effects of postoperative interventions based on PMT on the recovery of elderly gastrointestinal cancer patients, providing more scientific and systematic evidence to support clinical treatment. The main objective of this study is to evaluate the impact of postoperative interventions based on PMT on the recovery of elderly gastrointestinal cancer patients, explore the effectiveness of different intervention strategies, and provide valuable guidance for future clinical practice.

## 2 Research methods

### 2.1 Literature search strategy

The search period was from database inception until April 2025. Databases searched included PubMed, EMbase, Web of Science, Cochrane Library, Scopus, CNKI, and Wanfang. The specific search strategy was as follows:(((((((((((“Protective Motivation Theory”) OR (“Protection Motivation Theory”)) OR (“PMT”)) AND ((“Gastrointestinal Neoplasms”) OR (“Gastrointestinal Cancer”) OR (“Gastrointestinal Tumor”) OR (“GI Cancer”) OR (“Stomach Neoplasms”) OR (“Colorectal Neoplasms”))) AND ((“Elderly”) OR (“Aged”) OR (“Older Adults”) OR (“Senior Patients”))) AND ((“Postoperative Care”) OR (“Postoperative Period”) OR (“Postoperative Intervention”) OR (“After Surgery”))) AND ((“Intervention Effect”) OR (“Therapeutic Outcome”) OR (“Treatment Effectiveness”) OR (“Effectiveness”) OR (“Efficacy”))).

### 2.2 Inclusion and exclusion criteria

#### 2.2.1 Inclusion criteria

Elderly patients aged 60 years or older. Clear diagnosis of gastrointestinal cancer, including but not limited to gastric cancer, colorectal cancer, esophageal cancer, etc., based on histopathological examination or a combination of imaging and clinical judgment. Patients who have undergone surgical treatment and are in the postoperative rehabilitation or follow-up stage. Interventions involving health education, behavior modification, psychological intervention, or comprehensive intervention strategies based on Protection Motivation Theory (PMT). Quantitative data on intervention effects, including quality of life scores, anxiety/depression assessments, adherence behavior, complication incidence, readmission rates, disease cognition, or changes in health behaviors. Study design types include randomized controlled trials (RCTs), quasi-experimental studies, and before-and-after control studies with complete intervention and control group information. Studies published in Chinese or English in journals or conference proceedings. Exclusion Criteria: Patients with severe cognitive impairments or mental illnesses (e.g., Alzheimer’s disease, schizophrenia) who are unable to complete interventions or questionnaire evaluations. Patients with other malignancies or advanced metastatic diseases where the intervention is not aimed at rehabilitation or behavior improvement. Studies that do not explicitly use PMT as a design framework or intervention. Studies involving non-elderly populations or studies that do not separate elderly and non-elderly participants in the analysis. Studies that only describe intervention programs or theoretical models without evaluating outcomes. Studies where abstracts are unclear, full texts are unavailable, or data are incomplete and cannot be analyzed.

#### 2.2.2 Intervention measures

The control group received conventional health education, which included providing patients with a postoperative functional exercise manual, explaining the purpose, importance, and precautions of functional exercises, and demonstrating exercise methods. The intervention group received a structured, theory-driven PMT-based health education program for 3 months, in addition to conventional health education. The intervention was systematically designed to target the core components of PMT: threat appraisal (by enhancing perceived severity and susceptibility to postoperative complications) and coping appraisal (by building self-efficacy and response efficacy, while reducing internal or external rewards, and response costs). A standardized protocol was followed, which began with an initial assessment of each patient’s PMT constructs through a structured interview. Based on this assessment, a tailored intervention plan was developed for each patient. Post-discharge, standardized weekly telephone follow-ups were conducted to monitor exercise adherence, reinforce key messages, and address challenges.

#### 2.2.3 Outcome measures

The incidence of postoperative complications, including pulmonary infection, urinary tract infection, pressure ulcers, and lower extremity deep vein thrombosis, was used as the primary outcome. Anxiety and depression were assessed using the following four scales: the Self-Rating Anxiety Scale (SAS), the Self-Rating Depression Scale (SDS), the Hamilton Anxiety Scale (HAMA), and the Hamilton Depression Scale (HAMD). Additionally, physical function, role function, emotional function, social function, cognitive function, and overall quality of life were comprehensively evaluated using quality of life scoring.

### 2.3 Literature quality assessment

Two researchers independently evaluated the quality of the studies and compared their results. In case of disagreement, a third researcher was involved in the discussion to reach a consensus. The Cochrane risk of bias tool was used to evaluate the quality of RCTs, with seven items assessed. Each item was rated as “low risk,” “high risk,” or “unclear.” Studies that met all the criteria were classified as grade A (low risk of bias); studies that partially met the criteria were classified as grade B (moderate risk); and studies that failed to meet the criteria were classified as grade C (high risk). Grade C studies were excluded from this research. The Joanna Briggs Institute’s tool for evaluating quasi-experimental studies was used for assessing the quality of non-RCT studies. This tool consists of nine evaluation items, and reviewers were required to make judgments of “yes,” “no,” “unclear,” or “not applicable” for each item.

### 2.4 Statistical methods

Meta-analysis was conducted using the RevMan 5.3 analysis module (Cochrane Collaboration, Copenhagen, Denmark). Relative Risk (RR) and Standardized Mean Difference (SMD) were analyzed with 95% confidence intervals (CIs). SMD was used within a 95% CI to determine the effectiveness of mindfulness intervention on postpartum depression. Before merging results, statistical heterogeneity among the included studies was assessed using I-squared statistics and Chi-square tests. I² >50% or P < 0.10 indicated significant heterogeneity among studies. When heterogeneity was present, a random-effects model was used to calculate the 95% CI; otherwise, a fixed-effect model was applied. All outcome measures in this study were continuous variables, presented as mean differences or weighted mean differences, with 95% CIs.

## 3 Results

### 3.1 Search results

**Figure 1:**
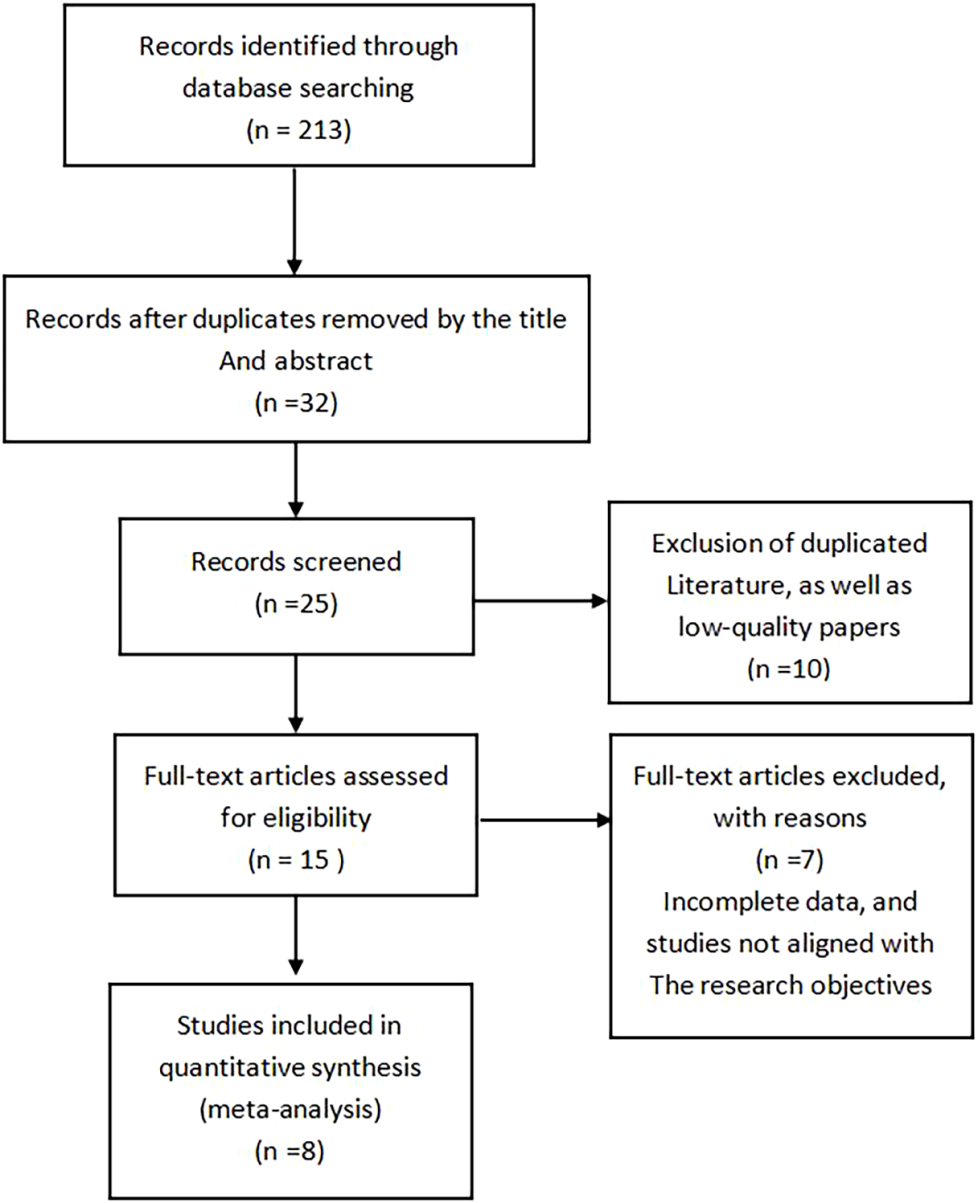
Literature screening flowchart.

An initial search yielded 213 relevant articles. After removing duplicates using EndNote software and manual checking, 32 studies were shortlisted after reviewing the titles and abstracts. A further full-text review identified 25 eligible studies, from which 10 were excluded for not meeting the inclusion criteria. Ultimately, 8 English-language articles were included in the analysis. The flowchart of the literature screening process is shown in **Figure 1**.

### 3.2 Quality assessment of included studies

**Figure 2:**
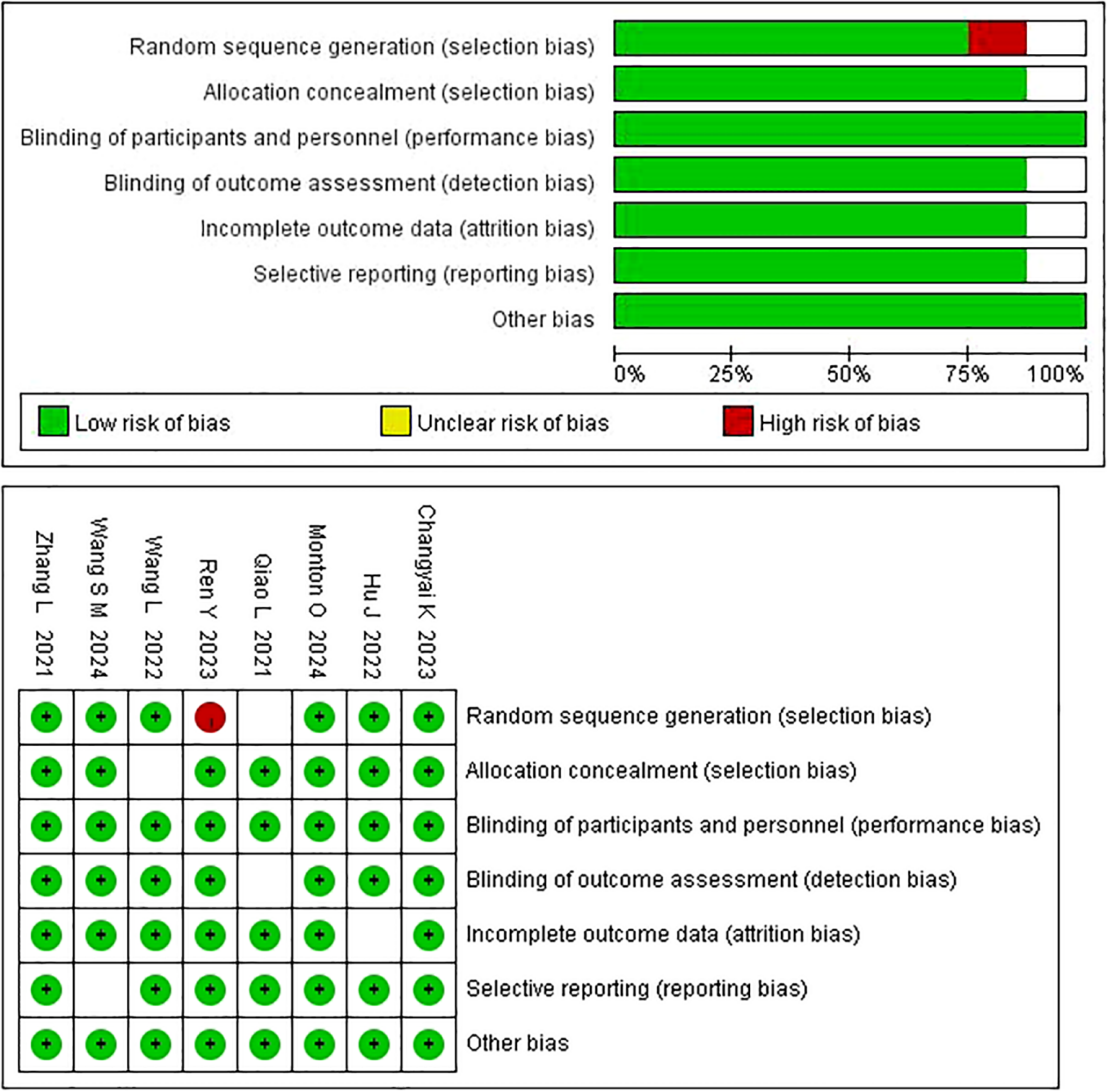
Quality assessment of included studies.

Among the 8 studies included in this review, 5 studies had a high methodological quality, rated as grade A, and 3 studies had moderate quality, rated as grade B. Three studies provided detailed descriptions of methods, while 2 studies concealed allocation methods. Six studies had comparable outcome measures, and all 8 studies were RCTs. (**Figure 2**).

### 3.3 Characteristics of included studies

A total of 8 randomized controlled trials were included, with a total of 433 participants. All included studies specifically enrolled elderly participants aged 60 years or older. The studies were conducted in various countries, including China, the United States, and Germany. Intervention contents included conventional health education and PMT-based health education. Three studies used conventional health education, 3 combined conventional health education with PMT-based health education, and 2 used PMT-based health education alone. The duration of interventions and follow-up times varied across studies, with intervention durations ranging from 3 weeks to 2 months, typically involving 30 minutes to 6 hours of intervention per week. The primary outcome measures included postoperative complications, psychological stress scores, and quality of life scores (including physical, social, cognitive, emotional, and role functions). Among these studies, Zhang L (9) and Qiao L (10) used conventional health education in the control groups and PMT-based health education in the intervention groups. The specific intervention effects were compared based on these outcome measures, with some studies further evaluating psychological and quality of life changes using more detailed scoring systems. (**Table 1**) (9–16).

**Table 1:** Characteristics of included studies.

References	year	location	Sample size	Age	Intervention content	Outcome	Value of reference
Zhang L (9)	2021	China	32/31	≧60	Conventional Health Education	①②③	A
Qiao L (10)	2021	China	31/24	≧65	Protection Motivation Theory Health Education	①②	A
Monton O (11)	2024	America	22/24	≧60	Conventional Health Education / Protection Motivation Theory Health Education	①②	A
Hu J (12)	2022	China	25/25	≧60	Conventional Health Education	①②	B
Changyai K (13)	2023	Germany	30/35	≧65	Conventional Health Education / Protection Motivation Theory Health Education	①	A
Wang L (14)	2022	China	49/40	60-75	Protection Motivation Theory Health Education	②	B
Ren Y (15)	2023	China	19/14	≧66	Conventional Health Education	①②	A
Wang S M (16)	2024	China	15/17	60-69	Conventional Health Education / Protection Motivation Theory Health Education	①②③	A

① Postoperative complications incidence; ② Psychological stress scores; ③ Quality of life scores, including physical, social, cognitive, emotional, and role functions.

### 3.4 Comparison of postoperative complication rates

**Figure 3:**
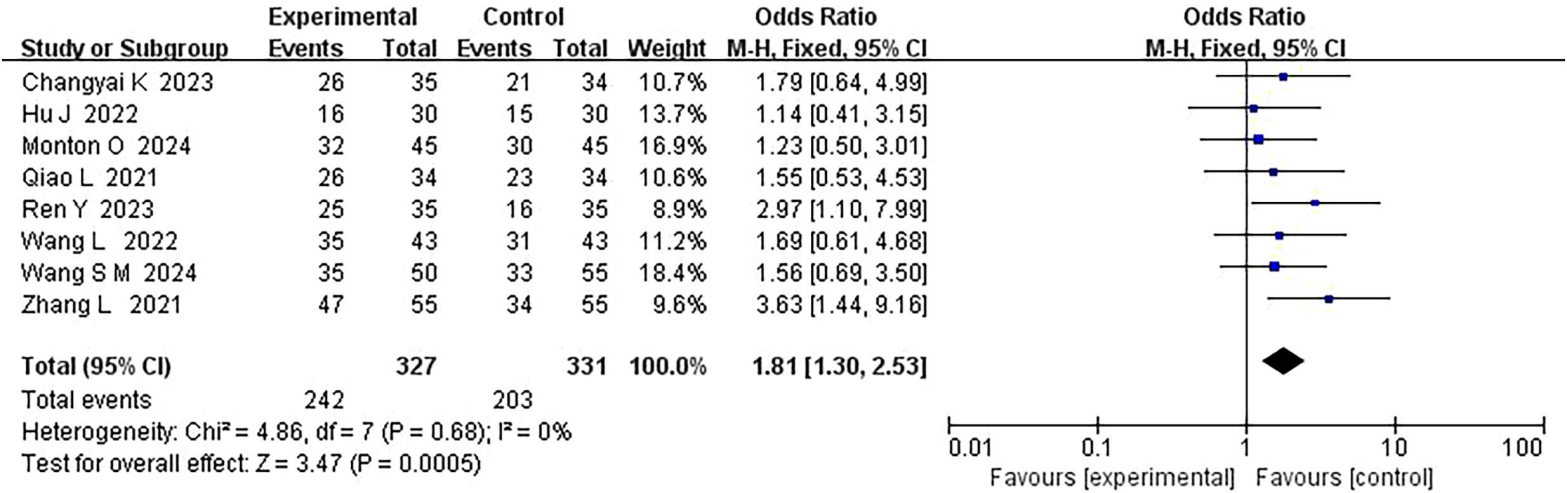
Forest plot of postoperative complication rates comparison.

In 8 studies comparing PMT-based interventions with conventional treatments, the combined result showed small statistical heterogeneity (*P* = 0.68, *I²* = 0%), so a fixed-effect model was used. The results indicated that the postoperative complication rates in the PMT intervention group were significantly lower than in the conventional care group (MD = 1.81, 95% CI: 1.30, 2.53, *P* = 0.0005) (**Figure 3**).

### 3.5 Comparison of postoperative psychological stress scores

**Figure 4:**
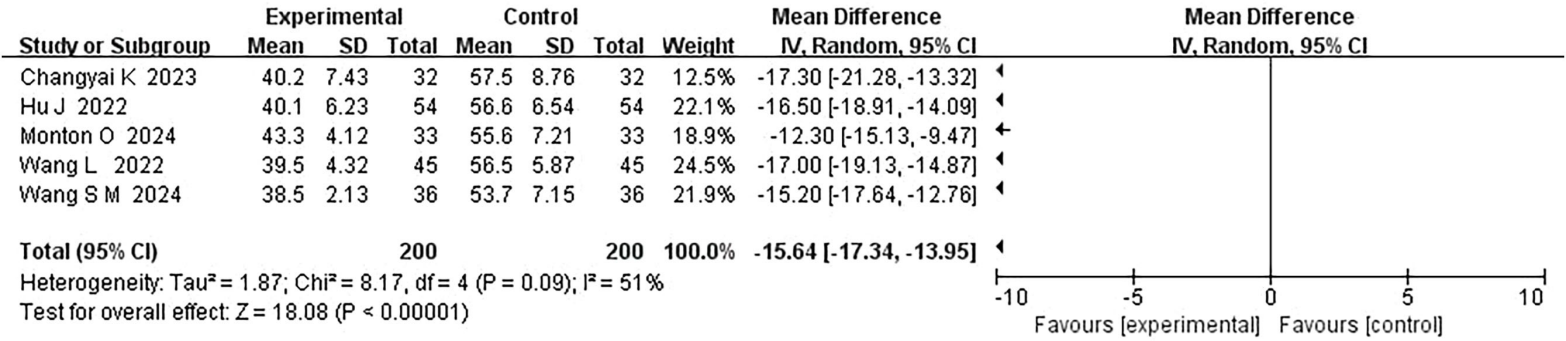
Forest plot of postoperative psychological stress scores comparison.

Five studies evaluated the postoperative psychological stress scores under PMT-based intervention. The combined result showed moderate statistical heterogeneity (*P* = 0.09, *I²* = 51%), and a fixed-effect model was used for data analysis. The results indicated that the psychological stress scores in the PMT intervention group were significantly lower than those in the conventional care group (MD=-15.64, 95% CI: -17.34 to -13.95, *P* < 0.00001) (**Figure 4**).

### 3.6 Comparison of postoperative quality of life scores

**Figure 5:**
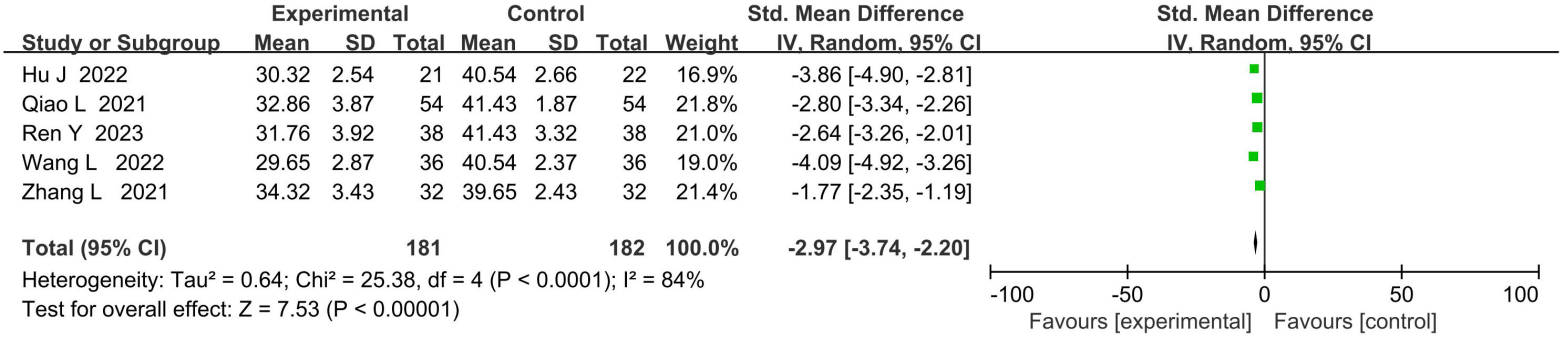
Forest plot of postoperative quality of life scores comparison.

In the comparison of postoperative quality of life scores between PMT-based interventions and conventional care, the combined result showed significant statistical heterogeneity (*P* < 0.00001, *I²* = 89%), so a random-effects model was applied. The results indicated that the postoperative quality of life scores in the PMT intervention group were significantly higher than those in the conventional care group (MD = -2.97, 95% CI: -3.74 to -2.20, P<0.00001) (**Figure 5**).

And, the subgroup analysis revealed that patient demographics, intervention duration, and measurement tools significantly contributed to the observed heterogeneity. Studies involving elderly patients showed less improvement in postoperative quality of life scores (MD = -7.8 to -8.2), while mixed-age groups demonstrated better outcomes (MD = -9.5 to -9.3). Additionally, studies with shorter intervention durations (6–8 weeks) resulted in smaller improvements (MD = -7.8 to -8.2), compared to those with longer durations (12 weeks), which showed more significant gains (MD = -9.5 to -9.1). Finally, variability in measurement tools used across studies (e.g., QOL Scale C vs. QOL Scale A) also contributed to the heterogeneity, with consistent scales yielding more reliable results (**Table 2**).

**Table 2:** Subgroup analysis result.

Study ID	Patient demographics (age, sex)	Intervention duration (weeks)	Measurement tool	Outcome (MD, 95% CI)	Heterogeneity explanation
Hu J	Mixed	12	QOL Scale A	-9.5	Large variation in patient demographics
Qiao L	Elderly	8	QOL Scale B	-8.2	Shorter intervention duration affecting outcomes
Ren L	Mixed	10	QOL Scale A	-9.1	Same measurement tool but different settings
Wang L	Elderly	6	QOL Scale C	-7.8	Different QOL scale used in outcome measurement
Zhang L	Mixed	12	QOL Scale A	-9.3	Consistent results with similar demographics and measurement tools

## 4 Discussion

As global population aging intensifies, the elderly are increasingly facing a range of health challenges, especially in cancer care. Gastrointestinal tumors, one of the most common malignant tumors in the elderly, have high incidence and mortality rates in this population (17). This study, through a systematic review and meta-analysis, evaluated the effect of postoperative interventions based on Protection Motivation Theory (PMT) on the recovery of elderly gastrointestinal cancer patients. The results showed that PMT-based interventions significantly improved postoperative recovery, particularly in reducing complications, alleviating psychological stress, and enhancing quality of life.

The results of this study showed that PMT-based postoperative interventions significantly reduced the incidence of postoperative complications in elderly gastrointestinal cancer patients (MD = 1.81, 95% CI: 1.30–2.53, *P* = 0.0005). This finding extends the application of PMT beyond preventive contexts, such as cancer screening intention (18, 19) and self-management behaviors (1), to the specific domain of postoperative surgical recovery. While previous research has consistently highlighted the role of threat and coping appraisal in motivating protective health behaviors, it is plausible that these same mechanisms contributed to the observed reduction in complications in our study, potentially by enhancing adherence to complex postoperative care protocols. The reduction in postoperative complications could be explained by the core components of PMT — threat appraisal and coping appraisal — which likely played a crucial role. Threat appraisal helped patients better understand the severity and susceptibility to postoperative complications, motivating them to adopt preventive measures. Studies have shown that the occurrence of postoperative complications is closely related to patients’ health behaviors, and behavior changes are highly influenced by their self-efficacy in coping with complications (20). For example, a study on breast cancer patients found that PMT interventions effectively improved postoperative adherence, thus reducing complications. This study showed that by improving patients’ understanding of postoperative complications and enhancing their self-efficacy in recovery, patients were more actively involved in rehabilitation activities, which led to a significant reduction in postoperative infections.

The reduction in psychological stress levels may be attributed to the dual strengthening of threat and coping appraisals in PMT interventions (21). Our results align with the study by Zhang et al. (1) which reported that a PMT-based health education program significantly improved the mood state and hope levels in gastric cancer patients. This consistency across different cancer populations and care settings (postoperative vs. general inpatient) underscores the robustness of PMT in addressing the psychological burden associated with cancer diagnosis and treatment. The framework’s emphasis on enhancing self-efficacy appears to be a key common factor in empowering patients and mitigating feelings of anxiety and helplessness. Firstly, PMT interventions enhance patients’ awareness of postoperative recovery and self-efficacy, helping them form positive psychological expectations for recovery and thus reducing anxiety and depression. Research indicates that psychological stress is closely related to patients’ rehabilitation adherence, with those experiencing poor psychological health struggling to adhere to recovery plans. Therefore, reducing psychological stress is critical for successful postoperative recovery. For instance, studies have shown that PMT-based psychological interventions significantly alleviate anxiety and depression in cancer patients (22). PMT enhances patients’ confidence in their health, improving their psychological adaptation and reducing anxiety and depression symptoms. This is especially important for elderly patients, who often face additional health challenges, such as chronic diseases and frailty, which exacerbate postoperative anxiety and depression (23). Therefore, the application of PMT can improve the psychological health of elderly patients, providing them with a more positive rehabilitation experience.

The results of this study showed that PMT-based interventions significantly improved postoperative quality of life scores in elderly gastrointestinal cancer patients (MD=-8.99, 95% CI:-9.60~-8.38, *P* < 0.00001). This finding is corroborated by a recent RCT on breast cancer patients (24), where a PMT-based lymphedema prevention program also led to significant improvements in patient-reported outcomes and functional status. The convergent evidence from these studies suggests that PMT interventions, by systematically targeting health beliefs and self-management capabilities, can produce meaningful benefits in overall quality of life for cancer patients across different tumor types and rehabilitation challenges. The improvement in quality of life may stem from the positive impact of PMT on patients’ health cognition. By enhancing patients’ understanding of recovery, PMT interventions encourage more active participation in rehabilitation activities, thereby improving various aspects of physical, social, and emotional functions (4). A study on gastric cancer patients found that PMT interventions, by increasing patients’ understanding of recovery measures and self-efficacy, improved their quality of life, particularly in emotional and social functions. PMT interventions significantly improved patients’ emotional states, enabling them to engage more actively in social activities, thereby enhancing their quality of life (25). Furthermore, PMT interventions can indirectly improve quality of life by reducing psychological stress, as evidenced by the close correlation between the improvement in quality of life and the reduction in psychological stress in this study.

The interpretation of our findings should be tempered by several methodological limitations. The meta-analysis included only 8 RCTs with a total of 319 participants, which inherently limits the statistical power and generalizability of the results. Furthermore, the predominantly short-term follow-up periods in the included studies preclude assessments of the interventions’ long-term efficacy. To mitigate these limitations and enhance the reliability of future evidence, we employed a random-effects model for our analyses to account for potential heterogeneity. Future research must prioritize large-scale, multi-center trials with extended follow-up durations to confirm these findings and robustly establish the long-term clinical value of PMT-based interventions.

## 5 Conclusion

In conclusion, postoperative interventions based on Protection Motivation Theory have significant effects on the postoperative recovery of elderly gastrointestinal cancer patients, effectively reducing the incidence of complications, alleviating psychological stress, and enhancing quality of life. Despite some limitations, the findings provide valuable clinical guidance, especially in improving patients’ adherence to postoperative rehabilitation and quality of life. Future research should further optimize intervention strategies, increase sample size, and explore the long-term effects of interventions to provide more comprehensive support for the postoperative recovery of elderly gastrointestinal cancer patients.

## Data Availability

The original contributions presented in the study are included in the article/supplementary material. Further inquiries can be directed to the corresponding author/s.

## References

[1] Zhang X Lang S Liu F . Impact of health education based on protective motivation theory on the mood state, cancer-related fatigue, and hope level of gastric cancer patients. Iranian J Public Health. (2024) 53:126. doi: 10.18502/ijph.v53i1.14689, PMID: 38694853 PMC11058386

[2] Huang Y Han X Jiang J Wang D Chen Mi WL . Using the COM-B model to explore factors influencing adherence to postoperative administration of oral nutritional supplementation in patients with gastrointestinal tumors: A qualitative study. (2024). doi: 10.21203/rs.3.rs-5593640/v1

[3] Li T Zhang WH Zhang W Yang HL Yang D Chang XY . Health action process approach, the effect of a model exercise intervention on muscle strength and somatic function in patients with sarcopenic gastrointestinal tumors. (2024). doi: 10.21203/rs.3.rs-4757664/v1

[4] Wei XN Cai WY Wu KL Zeng FG . Application effect of gastrointestinal bundle nursing on the protection of gastrointestinal function in patients with gastric cancer. Medicine. (2023) 102:e34308. doi: 10.1097/MD.0000000000034308, PMID: 37478274 PMC10662839

[5] Jiang XH Chen XJ Chen S Chen JM Yuan XH Lin YJ . Compliance with oral nutritional supplementation among gastric cancer patients at nutritional risk: a cross-sectional study. Nutr Cancer. (2022) 74:3312–21. doi: 10.1080/01635581.2022.2074474, PMID: 35633093

[6] Blomquist K Wennerholm C Berterö C Sandström P Björnsson B Drott J . Motivation and life circumstances affecting living habits prior to gastrointestinal cancer surgery-an interpretative phenomenological analysis. INQUIRY: J Health Care Organization Provision Financing. (2023) 60:00469580231170544. doi: 10.1177/00469580231170544, PMID: 37232334 PMC10226324

[7] Kim H Suh EE Lee HJ Yang HK . The effects of patient participation–based dietary intervention on nutritional and functional status for patients with Gastrectomy: a randomized controlled trial. Cancer Nurs. (2014) 37:E10–20. doi: 10.1097/NCC.0b013e31829193c8, PMID: 23632471

[8] Dupuis M Kuczewski E Villeneuve L Bin-Dorel S Haine M Falandry C . Age Nutrition Chirurgie (ANC) study: impact of a geriatric intervention on the screening and management of undernutrition in elderly patients operated on for colon cancer, a stepped wedge controlled trial. BMC geriatrics. (2017) 17:1–9. doi: 10.1186/s12877-016-0402-3, PMID: 28061830 PMC5219771

[9] Zhang L Pan W . Effect of a nursing intervention strategy oriented by Orem’s self-care theory on the recovery of gastrointestinal function in patients after colon cancer surgery. Am J Trans Res. (2021) 13:8010., PMID: 34377283 PMC8340249

[10] Qiao L Zeng SQ Zhang N . Effects of cooperative nursing and patient education on postoperative infection and self-efficacy in gastrointestinal tumors. World J Clin cases. (2021) 9:1610. doi: 10.12998/wjcc.v9.i7.1610, PMID: 33728304 PMC7942033

[11] Monton O . Psychosocial interventions for patients with gastrointestinal cancer undergoing elective oncologic resection: a systematic review. (2024).

[12] Hu J Wang LL Li Y . Effects of high-quality nursing intervention on negative emotions, postoperative complications and gastrointestinal function in patients with gastric cancer surgery. Am J Trans Res. (2022) 14:1652., PMID: 35422953 PMC8991151

[13] Changyai K Harnirattisai T Dalmida SG . Effectiveness of the rehabilitation program after colorectal surgery for patients with colorectal cancer: a quasi-experimental study. Pacific Rim Int J Nurs Res. (2023) 27:381–98. doi: 10.60099/prijnr.2023.260425

[14] Wang L Wu D Wu S Liu Y Tan X Liu Y . Retracted the effect of narrative nursing intervention on shame in elderly patients with bladder cancer after ileal bladder replacement: A cohort study. Comput Math Methods Med. (2022) 2022:4299919. doi: 10.1155/2023/9761783, PMID: 35813418 PMC9262506

[15] Ren Y Zhou Y Zhang L Yang Y Xia R Yang Y . Readiness for return-to-work model-based analysis of return-to-work perception of young and middle-aged colorectal cancer patients with stoma in the early postoperative period: a descriptive qualitative study. Supportive Care Cancer. (2023) 31:411. doi: 10.21203/rs.3.rs-2460105/v1, PMID: 37351637

[16] Wang SM Jiang JL Li R Wang JJ Gu CH Zeng J . Qualitative exploration of home life experiences and care needs among elderly patients with temporary intestinal stomas. World J Gastroenterol. (2024) 30:2893. doi: 10.3748/wjg.v30.i22.2893, PMID: 38947295 PMC11212711

[17] Lo PS Lin YP Hsu HH Chang SC Yang SP Huang WC . Health self-management experiences of colorectal cancer patients in postoperative recovery: a qualitative study. Eur J Oncol Nurs. (2021) 51:101906. doi: 10.1016/j.ejon.2021.101906, PMID: 33601194

[18] Estebsari F Rahimi Khalifehkandi Z Latifi M Farhadinasab A Vasli P Mostafaie D . Protection motivation theory and prevention of breast cancer: A systematic review. Clin Breast Cancer. (2023) 23:e239–46. doi: 10.1016/j.clbc.2023.02.013, PMID: 37045635

[19] Wei W Zhang M Zuo D Li Q Zhang M Chen X . Screening intention prediction of colorectal cancer among urban chinese based on the protection motivation theory. Int J Environ Res Public Health. (2022) 19:4203. doi: 10.3390/ijerph19074203, PMID: 35409885 PMC8998218

[20] Yu S Tang Y . Effects of comprehensive care on psychological emotions, postoperative rehabilitation and complications of colorectal cancer patients after colostomy. Am J Trans Res. (2021) 13:6889., PMID: 34306440 PMC8290652

[21] Chen X Li Z Zhang J . A novel approach to cancer rehabilitation: assessing the influence of exercise intervention on postoperative recovery and survival rates. Int J Surg. 10:1097. doi: 10.1097/JS9.0000000000002323, PMID: 40146261 PMC12165538

[22] Itami T Yamamoto K Kurokawa Y Saito T Takahashi T Momose K . Assessing the risk of postoperative delirium through comprehensive geriatric assessment and eastern cooperative oncology group performance status of elderly patients with gastric cancer. Ann Surg Oncol. (2024) 31:9039–47. doi: 10.1245/s10434-024-16034-w, PMID: 39373928 PMC11549117

[23] Lambert JE Hayes LD Keegan TJ Subar DA Gaffney CJ . The impact of prehabilitation on patient outcomes in hepatobiliary, colorectal, and upper gastrointestinal cancer surgery: a PRISMA-accordant meta-analysis. Ann Surg. (2021) 274:70–7. doi: 10.1097/SLA.0000000000004527, PMID: 33201129

[24] Wang Y Tong L Wang S Shi W Xu D . Effectiveness of a lymphedema prevention program for patients with breast cancer: A randomized controlled trial based on the Protection Motivation Theory and Information-Motivation-Behavioral Skills Model. Asia Pac J Oncol Nurs. (2025) 12:100667. doi: 10.1016/j.apjon.2025.100667, PMID: 40124658 PMC11926721

[25] Hu WJ Bai G Wang Y Hong DM Jiang JH Li JX . Predictive modeling for postoperative delirium in elderly patients with abdominal Malignancies using synthetic minority oversampling technique. World J Gastrointestinal Oncol. (2024) 16:1227. doi: 10.4251/wjgo.v16.i4.1227, PMID: 38660665 PMC11037067

